# New Drugs, Appliances, and Things Medical

**Published:** 1892-07-09

**Authors:** 


### NEW DRUGS, APPLIANCES, AND THINGS MEDICAL.

[All preparations, appliances, novelties, etc., of which a notice is desired, should be sent for The Editor, to care of The Manager, 140, Strand, London, W.C.]

### MELLIN'S FOOD.

We have received a sample of this food for notice. It consists of a light, brownish-yellow powder, with a very pleasant odour and sweet, malt-like flavour. It is soluble in water, and forms a pleasant tasting fluid, which on analysis gives abundant evidence of the presence of the various forms of sugar and sugar-like substances into which starch is converted by the action of diastatic ferments. Again, the presence of the soluble phosphates is easily demonstrated; in fact, what we have here is a pre-digested cereal food. Now we know that the stomach in a young infant is incapable of digesting starch, so this food does away with evil results of filling a baby's stomach with starchy food, which simply passes through its intestines unaltered, and may give rise to serious dyspeptic trouble. The food is not for use alone, but with cow's milk. By diluting cow's milk with a watery solution of the food, a fluid is formed which has a very close resemblance to human milk, and which presents for absorption the alimentary substances in such a form that the greatest quantity can be taken up in the very quick passage through the intestines which takes place in infants. We need not praise a preparation which has stood the test of time and experience so well; but we can honestly state that among the many infant foods on the market there are none which can beat this one. The inventor has carried out his ideas on physiological lines, and has succeeded in producing a food which, owing to civilisation, is an absolute necessity in the nursery.

